# Thoracic ultrasound evaluation and B-type natriuretic peptide value in elective cesarean section under spinal anesthesia

**DOI:** 10.1186/s13089-020-00158-7

**Published:** 2020-03-06

**Authors:** Luigi Vetrugno, Teresa Dogareschi, Rossella Sassanelli, Daniele Orso, Ludmilla Seremet, Lisa Mattuzzi, Sara Scapol, Alessandra Spasiano, Angelo Cagnacci, Tiziana Bove

**Affiliations:** 1 Anesthesia and Intensive Care Clinic, Department of Medicine, University of Udine, Via Colugna 50, 33100 Udine, Italy; 2 University-Hospital of S. M. Misericordia, Udine, Italy, 33100 P.le S. Maria della Misericordia n 15, Udine, Italy; 3 Hospital S. Vito al Tagliamento, Pordenone, Italy, Via della Vecchia Ceramica 1, 33170 Pordenone, Italy; 4 Gynecologic and Obstetric, Department of Medicine, University of Udine, Via Colugna 50, 33100 Udine, Italy

**Keywords:** Thoracic ultrasound, Cesarean section, B-type natriuretic peptide

## Abstract

**Background:**

Pregnancy-induced changes in cardiovascular status make women more susceptible to pulmonary edema. During cesarean section, to counterbalance the effect of hypotension caused by spinal anesthesia, anesthesiologists must choose between two fundamental approaches to maintain the hemodynamic state—intravenous fluids or vasopressors—and this choice will depend upon their particular opinions and experience. We aim to assess for any correlations between thoracic ultrasound A- and B-line artifacts, brain natriuretic peptide (BNP) levels, and the amount of intraoperative fluids administered.

**Results:**

From December 2016 to August 2018, at the University-Hospital of Udine, we enrolled 80 consecutive pregnant women undergoing cesarean section. We observed a statistically significant difference in the volume of fluids administered in the first 24 h (*p* = 0.035) between the patients presenting B-lines in at least one basal area of their thoracic ultrasound and patients with no evident B-lines (AUC 66.4%; IC 0.49–0.83). Dividing the population on whether their BNP levels were higher or less than 20 pg/mL, no statistically significant difference was revealed with regard to fluids administered in the first 24 h (*p* = 0.537).

**Conclusions:**

Thoracic ultrasound is a non-invasive and easy-to-use tool for detecting fluid intolerance in pregnant women undergoing cesarean section. BNP levels were slow to rise following the cesarean section and did not show any clear correlation with fluid volumes administered.

## Background

In the obstetric population, thoracic ultrasound (TUS) assessment and monitoring is a feasible and helpful diagnostic tool [1]. Importantly, in obstetric patients, the ultrasound exam is safe. The accuracy of TUS in revealing deviations from a healthy profile in pregnant women is very high: i.e., A-lines (indicative of an ordinary pattern), B-lines (indicative of an abnormal pattern), C-profiles (consolidation), A-lines without sliding (pneumothorax), etc. [2]. Furthermore, TUS expertise is easily attained following a concise training course for physicians and non-physicians alike [3]. In women with preeclampsia, pulmonary edema is associated with the appearance of B-lines, i.e., interstitial syndrome [4, 5]. Pregnancy causes an increase in the interstitial water compartment in multiple parts of the body, including the lungs [6, 7]. However, until now, despite the excellent diagnostic performance highlighted by the literature, TUS has never been used to evaluate this type of modification in pregnant women. During the cesarean section procedure, to counterbalance the effect of spinal anesthesia-induced hypotension, and depending on the knowledge and opinions of the individual anesthesiologist, the following hemodynamic strategies can be adopted: one based principally on the fluid administration and the other one addressed to vasopressor management [8–10]. In the present study, we hypothesized that changes in the TUS pattern and brain natriuretic peptide (BNP) level—a marker of congestive circulatory failure—could provide markers of fluid overload [11–13]. The aim of the present study was, therefore, to investigate whether a relationship exists between TUS, BNP, and the volume of intraoperative fluids administered (in the 24 h after cesarean section).

## Methods

This observational, single-center, retrospective study was carried out at the University-Hospital of Udine (Italy)—a level-3 maternity center performing approximately 300 cesarean sections per year. Ethical approval was provided by the Regional Ethics Committee of Friuli-Venezia-Giulia on 5th February 2019, ID #2637, for principal investigators Dr. V.L. and Dr. T.D.

Before performing the statistical analysis, the trial was registered at clinicaltrials.gov with NCT number 03851679 on 20th February 2019. Given the retrospective design of the present study, patient consent was waived, but the European Privacy Regulation 2016/679 on General Data Protection Regulation (GDPR) was respected. Only pregnant women aged 18 years or over undergoing elective cesarean section in the absence of hypertensive disorders at gestational age no less than 37 weeks were included in the study; all women presented a physical status of 2 according to the American Society of Anesthesiologists classification system. Exclusion criteria were: emergent cesarean section, a history of pre-existing respiratory or cardiac disease, twin pregnancy; refusal to participate in the study.

In the operating theater, non-invasive standard monitoring with heart rate, non-invasive arterial pressure, and peripheral oxygen saturation were used (Philips, IntelliVue MX 700, Milano, Italy). For subarachnoid anesthesia, the patient was placed on their left side and a 25 G Sprotte needle (Pajunk Sprotte 25G × 90 mm, Geisingen, Germany) inserted by the resident physician or the consultant anesthesiologist following a failed first attempt. Following cephalorachidian fluid spread, 2 mL hyperbaric bupivacaine 0.5% (10 mg in total) (Bupivacaine Fresenius Kabi 5 mg/mL, Verona, Italy) plus 0.1 mg morphine (Morfina Cloridrato 10 mg/1 mL Monico, Venezia, Italy), as long-term analgesia, were injected. Immediately afterward, the patient was returned to the supine position, and the uterus pushed to the left. Arterial hypotension was controlled using a 3 mL/kg fluid challenge of crystalloids (RA Baxter Viaflo 1000 mL, Monselice-Padua, Italy) or an ephedrine bolus (ephedrine 3 mg/mL, 10 mL Aguettant, Venezia, Italy). A bladder catheter was placed in the ward before coming into the theater. The surgeon then performed a Pfannenstiel skin incision with the layered opening of the abdomen and celiotomy. The bladder–uterine fold was opened and the bladder detached at the bottom. A transverse incision was made on the lower uterine segment and the uterine breach opened by digital divulsion followed by fetal extraction [14].

Patient venous access was obtained before entering the operating theater, and a blood sample taken for the first BNP dosage (*T*0). Postoperative BNP was measured at 6 (*T*1) and 24 h (*T*2) after surgery. Blood was collected in EDTA and lithium heparin vacutainer tubes, and sent immediately for analysis to the laboratory (through a specialized internal transport service) where BNP concentrations were assayed using a Bayer ADVIA Centaur-TM. Test values were not known in real-time, and the physician and patients were blind to their results.

Thoracic ultrasound was performed in a semi-recumbent position using a SonoSite machine (S-Fast, Ltd.-Alexander House, Wilbury Way, Hitchin, Herts, SG4 OAP, UK) with a convex or linear probe and the “eight-region technique” as defined by the “International Consensus Conference on Lung Ultrasound” [15]. The probe was placed perpendicular to the ribs to visualize the “bat sign”, then translated into the oblique position along the intercostal spaces. A healthy lung was described as “A-Line with sliding”; an abnormal lung was described as “A-Line without sliding” (A’); a “B-Line” lung was one with three or more B-Lines per region; a coalescent B-Line was considered a “white lung”; and a “C-profile,” i.e., consolidated parenchyma and anechoic space, indicated pleural effusion [15]. The exam was performed at *T*0 (before cesarean section, immediately after obtaining the first BNP sample), at *T*1 (6 h after cesarean section), and *T*2 (24 h after surgery).

The perioperative data collected also included details regarding the duration of surgery; the spinal thermal level at discharge from the operating room, blood loss (mL); intraoperative diuresis (mL); and administered crystalloids (mL). Follow-up was planned until discharge.

The quantitative variables are presented as means and standard or median deviations plus the range according to their distribution. Qualitative variables are expressed as absolute and relative frequencies. The relative frequency and 95% confidence interval of patients with an altered TUS pattern is also shown. The quantitative variables of the positive TUS and negative TUS group are compared using the Mann–Whitney non-parametric test for independent samples, taking into account the distribution of the variables. The Chi-square test is used to estimate the association between qualitative variables. We also assessed the distribution of the relative increase in BNP ([logBNP2 − logBNP1]/BNP1) at the different time points (*T*0, *T*1, and *T*2) and applied ANOVA to assess for any statistically significant differences between the three groups. We used Hommel’s method to correct for multiplicity [16].

All statistical analyses were carried out using SPSS Statistical Package version 22 (Armonk, NY: IBM Corp) and R-Cran ver. 3.4.2 language and environment for statistical computing (R Core Team; R Foundation for Statistical Computing, Vienna, Austria, http://www.R-project.org).

Our study was an observational retrospective study that intends to estimate the relative frequency of pregnant women with an elective cesarean section presenting sub-clinical changes in the TUS pattern in the peripartum period. Data of a total of approximately 80 patients were available. Having no previous information on the prevalence of pregnant women with elective cesarean section presenting sub-clinical changes in the TUS picture in the peripartum period and assuming that this prevalence settled at around 50% (cases with maximum variability), 80 patients would allow us to obtain an estimate of this prevalence with an accuracy of 10%, which for this study was considered acceptable from the clinical point of view.

## Results

**Fig. 1:**
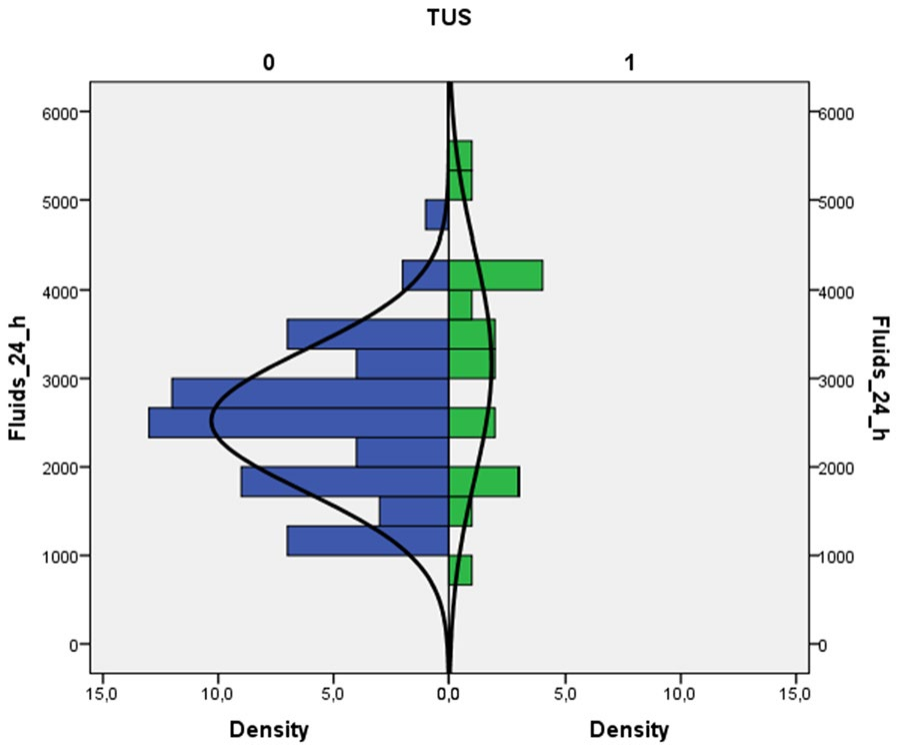
Distribution of the amount of fluids administered in the first 24 h after cesarean section, in the study population divided on the presence or absence of at least one area with B lines on thoracic ultrasound

**Fig. 2:**
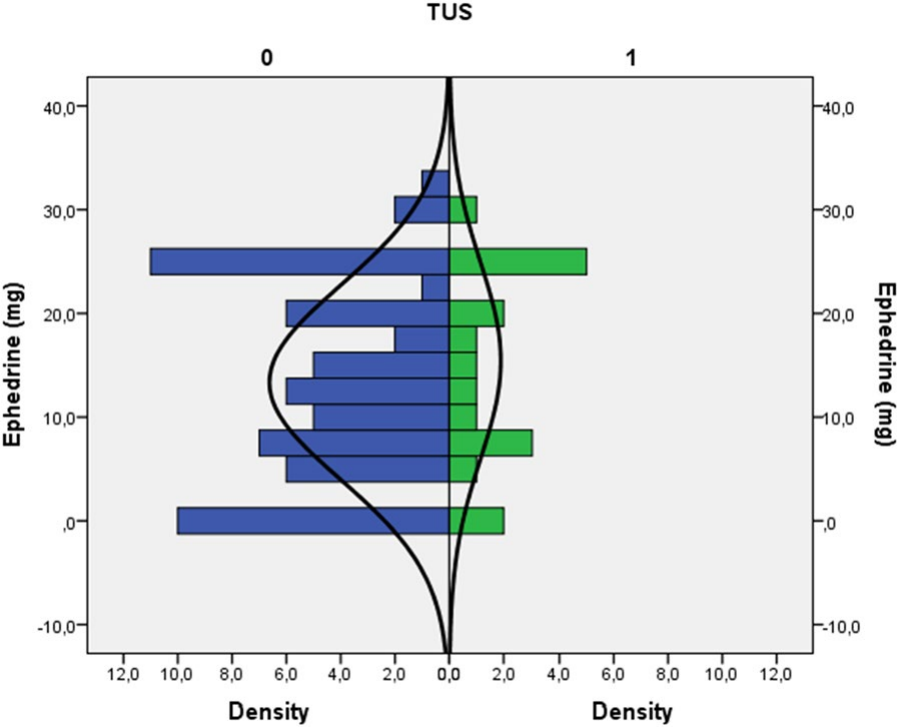
Distribution of the amount of ephedrine in the study population divided for the presence or absence of at least one area with B-lines

**Fig. 3:**
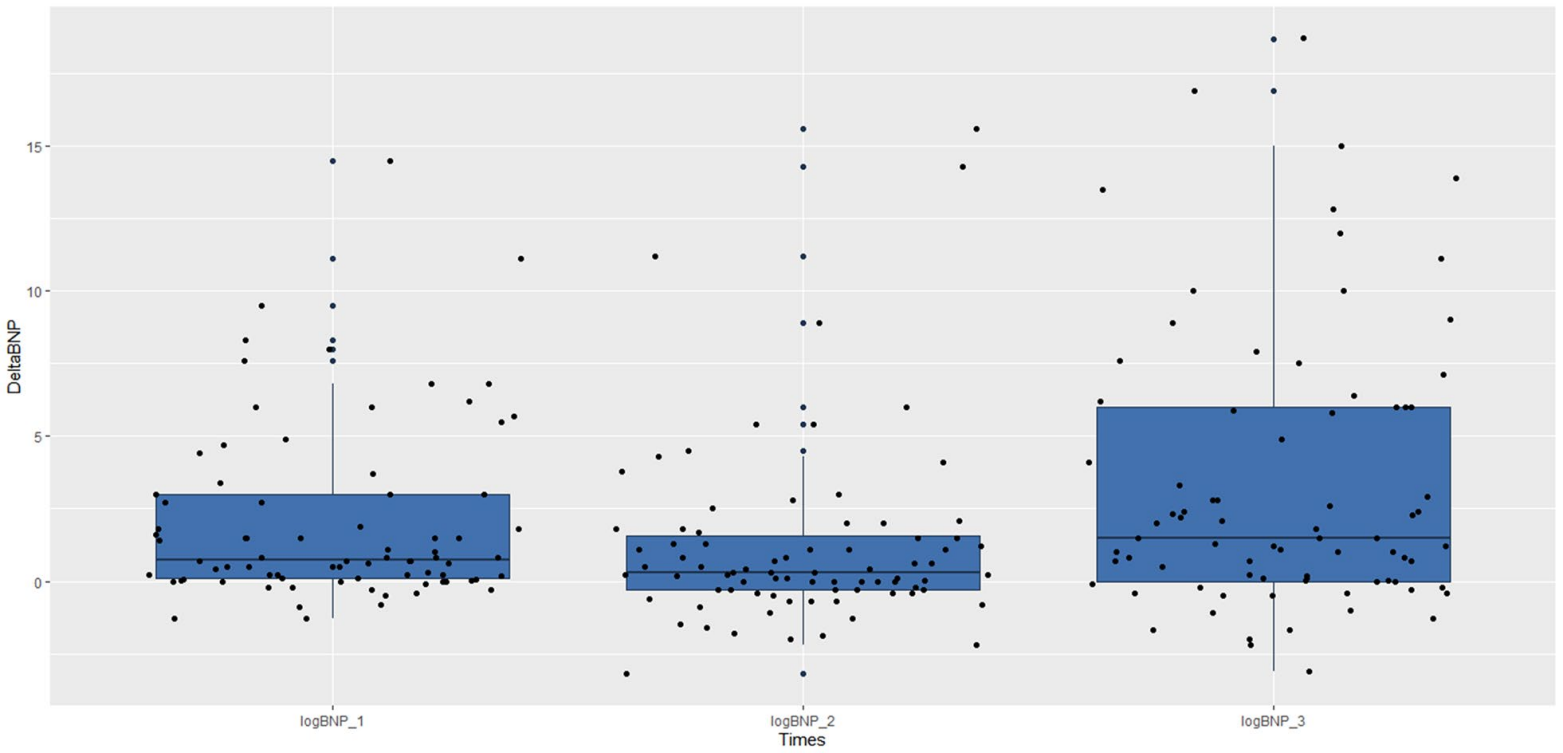
Comparison between the relative BNP increases between *T*0–*T*6; *T*6–*T*24 and *T*0–*T*24

From December 2016 to August 2018 at the University-Hospital of Udine, 84 pregnant women were approached to participate in the study. Of these, 80 were enrolled; in the remaining 4 cases, 3 had incomplete data, and in 1 case, the patient asked to leave the measurement after the first TUS evaluation. In all cases, patients underwent a cesarean section under spinal anesthesia. No further missing or lost data were encountered. Considering the sensitivity of TUS for the primary outcome of 50%, this sample size obtained an accuracy of 10%. The study population’s characteristics are described in Table 1. Briefly, mean patient age was 34 years, mean BMI 28.5, and mean gestational age 38 weeks with no previous history of cardiac or respiratory disease. During the study period, 240 TUS were performed. The greatest number of actual TUS exams, defined as the presence of at least one basal area presenting three or more B lines (see “Methods” section), was observed 6 h after cesarean section, being observed in 10 patients (12.5%). By analyzing the distribution between the two groups of the sample population (i.e., positive TUS vs. negative TUS) using the Mann–Whitney test, we were able to identify a statistically significant difference in the amount of fluids administered in the first 24 h (740.50; SE 86.63; *p* = 0.035) (Fig. 1). A receiver operating curve (ROC) was plotted for a total fluid balance at 24 h greater than 1000 mL and a positive TUS outcome. The best combination of sensitivity and specificity was for > 2500 mL fluids (sensitivity = 67% and specificity = 53%) with an area under the curve (AUC) of 66.4% [SE = 0.09; the 95% confidence interval (95% IC) ranged from 0.49 to 0.83]. We did not observe any correlation between the use of vasopressors and the amount of fluids (Fig. 2). Although the exact duration of each TUS exam was not recorded, in all cases, it required no more than just a few minutes to perform. During the study period, a total of 240 BNP tests were obtained. BNP values show a tendency to increase between time zero (*T*0) and 6 (*T*1) and 24 h (*T*2). As regards the relative increase between the three analysis times (*T*0-*T*1; *T*1-*T*2 and *T*0-*T*2 h), we found a statistically significant difference between the last two time comparisons (respective p-values: 0.437, 0.045 and 0.007). The BNP comparisons are shown in Fig. 3. When we divided the population based on a BNP value at 24 h (*T*2) greater than 20 pg/mL, we did not observe a statistically significant difference with regards to fluid balance in the first 24 h (Mann–Whitney test: 597.50; SE 93.90; *p* = 0.537). A statistically significant correlation was observed between the median BNP value at the time of delivery and 24 h (Chi-square = 4.09; *df* = 1; *p* = 0.04); while no correlations were observed between BNP and TUS, or between BNP and the volume of fluid administered (Table 2).

**Table 1:** Descriptive statistics of the sample population (*n* patients = 80)

	Mean	St. dev.
Descriptive features		
Age (years)	34.10	5.71
Gestational age	38.66	0.67
Number of pregnancies	0.86	1.24
Pre-pregnancy weight (kg)	63.76	10.97
Weight at the delivery (kg)	76.68	11.22
BMI pre-pregnancy	23.70	4.43
BMI at term	28.49	4.53
ΔBMI	20.94	8.46
Duration of delivery (min)	57.60	14.73
Max thermic level	3.99	1.00
Thermic level at the discharge	5.26	1.24
Duration of recovery (days)	3.84	0.95
Hemodynamic effects		
Blood loss (mL)	693.13	409.29
Crystalloids (mL)	1306.88	327.22
Ephedrine (mg)	13.82	9.35
Neosynephrine (mcg)	1.31	11.19
Diuresis at 24 h (mL)	1801.46	897.70
Total amount of administered fluids in 24 h (mL)	2665.94	962.41
Total balance mL (mL)	1478.23	1004.12

*BMI* body mass index

**Table 2:** Correlation (Chi-square values) between BNP, TUS and amount of fluids in the first 24 h

	TUS	BNP at time 0	BNP at 24 h	Fluids > 1000 mL
TUS		0.22 (*df* = 1; *p* = 0.64)	0.48 (*df* = 1; *p* = 0.49)	0.87 (*df* = 1; *p* = 0.35)
BNP at time 0	0.22 (*df* = 1; *p* = 0.64)		*4.09 (df* = *1; p* = *0.04)*	0.87 (*df* = 1; *p* = 0.35)
BNP at 24 h	0.48 (*df* = 1; *p* = 0.49)	*4.09 (df* = *1; p* = *0.04)*		0.54 (*df* = 1; *p* = 0.46)
Fluids > 1000 mL	0.87 (*df* = 1; *p* = 0.35)	0.87 (*df* = 1; *p* = 0.35)	0.54 (*df* = 1; *p* = 0.46)	

*TUS* thoracic ultrasound, *BNP* brain natriuretic peptide

## Discussion

The main findings of our study are twofold: first, during the perioperative period of the cesarean section with subarachnoid anesthesia, the TUS pattern showed a parabolic representation with maximum expression 6 h after surgery; second, BNP levels followed a linear trend with maximum values at 24 h. Based on these results, the TUS result obtained does seem to correlate with the amount of fluids administered to the patients in the first 24 h. On the contrary, BNP values were seen to increase more gradually over time and did not demonstrate any clear correlation with the volume of fluids administered. These findings suggest that TUS is more sensitive than the BNP value for determining fluid intolerance in the population studied. However, pregnancy is accompanied by many different physiological changes in the metabolic, endocrine, respiratory and hemodynamic systems [6, 7, 17]. As the uterus becomes bigger, it compresses the abdominal vessels and reduces venous return to the heart; cardiac output and blood volume are both increased, and systemic vascular resistance and blood pressure are reduced, while thoracic hydrostatic pressure goes up [18]. Together, these changes can lead to an increase in vascular permeability and induce an interstitial syndrome in the lung that predisposes pregnant women to pulmonary edema, particularly during severe preeclampsia [4, 6, 7, 18].

Neuraxial anesthesia, as used in cesarean section, can induce hypotension by vasodilation, which is usually corrected with fluidic therapy and vasopressors [19]. Furthermore, due to the resolution of spinal anesthesia and fluid redistribution, obstetric patients have a greater risk of fluid overload [20]. Many studies have tried to address the management of hypotension during spinal anesthesia for cesarean section [21–23]. Given the morbidities associated with maternal hypotension, affecting both mother and child, a recent international expert consensus recommended the use of vasopressors to maintain systolic arterial pressure to ≥ 90% of baseline [24]. How this may reduce the administration of fluids and, therefore, at least potentially, reduce fluid overload is still a matter of debate. On the one hand, physicians must try to reduce hypotensive episodes, but on the other, the negative effects of excess fluids (especially crystalloids) are well documented [9, 10, 25].

In pregnant women, a different strategy is adopted to monitor fluid management: Burlingame et al. showed that in healthy women the levels of BNP, or its precursor N-terminal-pro-BNP, as well as left atrial and left ventricular volumes all increase within 48 h after partum and are associated with diastolic dysfunction [26]. Ortner et al. found an association between raised BNP levels and echocardiographic markers of impaired systolic and diastolic function with late-onset in severe preeclampsia women [27]. In our study, the kinetics of BNP does not seem to be particularly sensitive in detecting any fluidic overload, even though BNP levels showed an increase in the first 24 h after surgery. On the contrary, a previous study showed TUS to be highly sensitive in its quantification of extravascular lung water in critically ill patients [28].

In a recent observational study, Arbeid et al. demonstrated the efficacy and the feasibility of TUS as a diagnostic tool during pregnancy through their study of 150 parturients between the 36th and the 38th gestational week [29]. This study also showed that the most common ultrasound pattern in pregnant women is the A-profile. On the contrary, Krawczyc et al. reported that lung ultrasound (LUS) was abnormal in at least one region—showing three or more B-lines—in 21% of their population of women during labor [30]. Although this was a small study involving just 24 women with uncomplicated labor, it reminds us that a basal examination is of paramount importance for detecting changes during labor and for being able to identify the correct therapy. Zieleskiewicz et al. first demonstrated that TUS interstitial syndrome in preeclampsia women could be better detected using ultrasound than clinical signs [4] and that the accuracy of TUS in detecting interstitial edema at a pre-clinical stage allowed adequate fluid resuscitation in patients with a high risk of alveolar pulmonary edema [4, 5]. In our study, we provide evidence of a significant correlation between the volume of fluids given during the first 24 h post-cesarean section and the detection of at least one basal lung region showing more than three B lines. Considering the speed at which TUS results are obtained and the fairly good sense that this method demonstrates, TUS could prove to be a handy tool for the early detection of “fluid intolerance” in the post-partum period.

A particular strength of this study lies in the fact that our resident physicians in anesthesia extensively use TUS in the intensive care unit and complete a year-long internal course on its use before moving to the obstetrics department; thus, their proficiency level is particularly high [31]. A weakness, however, comes with the fact that it is a retrospective single-center observational study. This study is the first to show TUS as a potent tool in the detection of lung intolerance [32], paving the way for future opportunities in the application of TUS in the surgical setting. At present, this study does not achieve a very high degree of evidence, and last but not least, we describe B-pattern only in one basal lung zone [33]. Furthermore, we are aware that the correlation between the presence of at least one basal lung region with a B-pattern and the presence of an interstitial syndrome is not so close. Other conditions, such as position atelectasis, or diseases, can mimic this pattern. However, the periodic and sequential evaluations of our patients allowed us to exclude the above conditions in most cases. In any case, since it is a pilot study, further assessment in the same population must be carried out.

## Conclusions

In conclusion, our study shows that TUS abnormalities (B-lines) correlate in the first hours with the volume of fluids given during the perioperative period of cesarean section, whereas BNP levels were slow to increase and did not shown any clear correlation with administered fluid volumes. Thoracic ultrasound is a non-invasive and easy-to-use tool that holds great promise for the detection of fluid intolerance in pregnant women. A multi-center study is now warranted to confirm our results.

## Data Availability

All data and materials are available if requested.

## Abbreviations

TUS: Thoracic ultrasound
BNP: Brain natriuretic peptide
EDTA: Ethylene-diamine-tetra-acetic acid
ANOVA: Analysis of variance
BMI: Body mass index
ROC: Receiver operating characteristic
AUC: Area under the curve
SE: Standard error
IC: Interval of confidence
